# Veterinary Progress a Gain to Science

**Published:** 1890-03

**Authors:** Cornelius M. O’Leary


### EDITORIAL.

### VETERINARY PROGRESS A GAIN TO SCIENCE.

THE chief tentative of modern intellectual activity has been to effect a continuity of all the sciences by classifying them on a strictly philosophical basis. During its earlier period of development each science pursued its course independently of sister sciences, and vainly strove to reach its goal by keeping within the technical limits of its subject-matter. But as it approached maturity the lines of divergence widened till they coalesced with other lines, and then it was seen that all the sciences possessed an essential unity, and that, while they tended more and more to heterogeneity in their results, they partook of a correspondingly homogeneous origin.

When scientific men of a philosophical turn clearly perceived this truth they addressed themselves to the task of finding a common groundwork for the various departments of science, and this aim was sought after in a variety of ways. Two attempts at such classification alone deserve mention, the one as having enlisted the efforts of some of the most eminent scholars of the early part of the century, and the other as confirming at once and receiving proof from the doctrine of evolution.

Comte, in France, and John Stuart Mill, in England, were the first to attempt a classification of the sciences on a historical basis, and though they expended much labor and ingenuity in the endeavor they reasoned from mistaken premises and consequently failed. When Darwin proclaimed to the world his fruitful theory of the common genesis of organized beings, he opened the way to a truly philosophical classification of the sciences, and even afforded to Spencer and Huxley the cue to their application of the principle of evolution to every problem within the range of historical, social, political, and moral philosophy. But, holding only to what the doctrine of evolution has firmly established, we find that physiology has contributed more substantial results to this end than any other special branch of science. Darwin's immortal work on the "Origin of Species" derives its chief value from the amount of physiological research it embodies, and from the extended range of his inquiries in that direction. Up to his time comparative physiology had made but little progress and the functions of life in the lower animals were regarded as quite subordinate to similar functions in man.

The impetus given by Darwin to this branch of science was felt in every department, for organic chemistry, ethnology and philology assumed new phases of interest in the light which comparative physiology shed upon them. But, while too much credit cannot be given to Darwin, and, at a later period and to a greater extent perhaps, to Romanes, it remains true that these inquirers were governed in their researches by a desire to make their discoveries contributive to their theories. Thus, while those ascertained results were so many valuable acquisitions to comparative physiology, they were regarded by those who brought them to light as valuable in so far only as they served a purpose, but were not incorporated into a general system of physiology. The motive for such incorporation did not exist, since no practical consequences depended upon it. It was only when the quickening of a scientific impulse was felt by men whose business in life it was to know and apply the principles of physiology in the lower organisms that the science of comparative physiology could be said to have found a permanent basis and taken root in a fruitful soil. When veterinary medicine left the ruts of empiricism and asserted itself as a science in all respects equal to that which boasted Hippocrates as its father, then did comparative physiology and pathology assume the character of a stable science with special reference to immediate consequences, and then were ardent researches instituted into the anatomy and physiology, the histology and functions of every species throughout the animal kingdom. These researches were systematically conducted and had a definite end in view. They were intended as the means of gathering together the materials for a new science, of furnishing the groundwork of a profession new in reality if not in name.

Already, indeed, the great principles of general physiology had been established, and these lent valuable assistance to the speedy development of veterinary science ; but had not the new school of veterinarians been men of a truly scientific turn and animated by the same spirit which prompted all sincere inquiries in their work, viz. : a love of truth and a passion for knowledge, these general principles would have found no application outside of the human subject, and veterinary science would be still relegated to the domain of quackery and empiricism. But men like Chauveau, Wesley Mills, Flemming, Liautard and many others infused the true scientific spirit into their work and threw floods of light on the anatomical and physiological peculiarities not only of the whole group of domestic animals, but incidentally elucidated some of the most vexed problems which Lamarck and Darwin set before themselves to solve.

Their work was earnest and well directed, and as a consequence the whole body of the new professional science received a strong and wholesome impulse. But while this desirable result was being accomplished a grander science, because the resumé of all the sciences, was receiving untold and unexpected advantages from the new and unlooked-for line of labor. The whole group of sciences, comprised within the scope and operation of evolutionary principles, was grandly aided by the labors of skilled and conscientious workers in the field of comparative physiology and anatomy. They brought together and systematized the fragmentary odds and ends of individual and independent inquiries and made these subservient to the noble purpose of the grandest system of philosophy of modern times. This result may not have been altogether foreseen by the pioneers of recent veterinary science, but none the less is the truth obvious that the patrons and advocates of modern veterinary medicine have contributed an unusual share to the advancement and the hopes of evolution. This, then, is where veterinary science stands. It has unfolded for itself a glorious future and it has contributed largely to the advancement of the pivotal idea of evolution.

CORNELIUS M. O'LEARY, M.D., Ph.D., LL.D.

